# Influence of dietary intake and eating patterns on reactive hypoglycemic events in patients postesophagectomy: A prospective observational study using continuous glucose monitoring

**DOI:** 10.1002/ncp.70022

**Published:** 2025-09-07

**Authors:** Rachel O'Kelly, Elaine Quigley, Katie Byrne, Mistura A. Kareem, Niamh Ni Mhaoinigh, Paul Healy, Michelle Fanning, John V. Reynolds, Claire L. Donohoe, Suzanne L. Doyle

**Affiliations:** ^1^ School of Biological, Health and Sports Sciences, Technological University Dublin Dublin Ireland; ^2^ Trinity St James's Cancer Institute Dublin Ireland; ^3^ Department of Clinical Nutrition & Dietetics Naas General Hospital Naas Ireland

**Keywords:** adult, diabetes, gastroenterology, life cycle, nutrition support practice, nutrition support teams, oncology, rehabilitation, research and diseases, surgery

## Abstract

**Background:**

Esophagectomy causes anatomical changes that can lead to rapid food transit and reactive hypoglycemia (RH). Patients are advised on eating patterns postesophagectomy to prevent RH, but its true incidence and the impact of dietary recommendations remain under‐researched.

**Materials and Methods:**

Individuals >12 months postesophagectomy were recruited from the National Centre for Oesophageal and Gastric Cancer at St James's Hospital in Dublin, Ireland. Over 7 days, continuous glucose monitoring (CGM) captured glucose readings, with food and symptom diaries documenting dietary intake and symptoms. The nutrition composition of meals was calculated, and food diaries were coded for the following eating patterns: leaving >3 h between meals, simple sugars with meals, fluid with meals, and alcohol with meals. Data analysis compared eating patterns preceding asymptomatic and symptomatic RH events. In all cases, *P* < 0.05 was considered statistically significant.

**Results:**

Thirty‐two participants completed the study, with 21,504 glucose readings and 1276 meals analyzed. CGM identified 226 meals (17.7%) followed by RH events, 19 of which were symptomatic. Meals associated with RH events were higher in carbohydrate (35.3 g vs 31.7 g; *P* = 0.036), fiber (4.11 g vs 3.15 g; *P* = 0.020), and sugar (12.65 g vs 10.96 g; *P* = 0.048). Leaving >3 h between meals and consuming alcohol with meals also increased RH risk. Nutrient composition and eating patterns did not differentiate symptomatic from asymptomatic RH events.

**Conclusions:**

Total carbohydrate content and specific eating patterns appeared to significantly influence RH incidence, with most RH events being asymptomatic. CGM may serve as a useful adjunct to dietary interventions in the management of RH in patients postesophagectomy.

## Graphical abstract

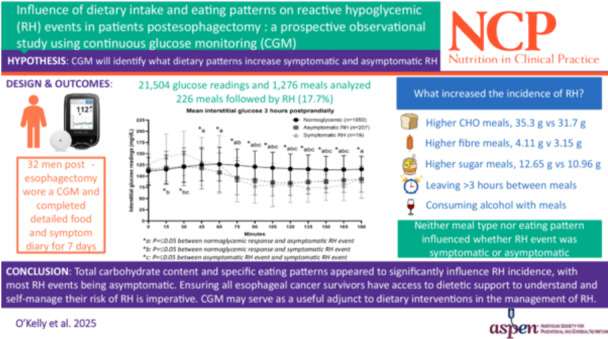

## INTRODUCTION

Esophageal cancer (EC) is the eighth most common cancer worldwide,^1^ and curative treatment includes esophagectomy.^2^, ^3^ This procedure can have different approaches, including Ivor Lewis, transhiatal, and left thoracoabdominal^2^ with pyloroplasty and gastric pull‐up.^4^, ^5^, ^6^, ^7^ The anatomical changes that occur after esophagectomy may promote disordered digestive function because of exocrine pancreatic insufficiency, bile acid malabsorption, small intestinal bacterial overgrowth, dumping syndrome, and reactive hypoglycemia (RH).^3^ Indeed, malabsorption and symptoms with nutrition impact are commonly reported in EC survivors, giving rise to weight loss, malnutrition, and reduced quality of life.^3^, ^8^, ^9^, ^10^, ^11^, ^12^ With EC survivorship rates increasing,^13^, ^14^ there is a need to explore the management of these postoperative side‐effects to improve outcomes in these patients.

The reconstruction of the esophagus following esophagectomy can lead to altered gastrointestinal transit and gut hormone signaling, giving rise to RH.^15^ This occurs 1–3 h postprandially and is associated with neuroglycopenic symptoms, which include, dizziness, faintness, cold sweats, cramps, hunger, and, in some cases, fatigue and nausea.^16^, ^17^ Although dumping syndrome has been reported to occur in up to 74% of patients postesophagectomy,^18^ there is limited information on the specific rates of RH, that is, “late dumping,” with a study by Anandavadivelan et al^19^ reporting a prevalence of 15%.

RH can be classified as symptomatic and asymptomatic. Indeed, many patients who experience recurrent RH can have defective glucose counter regulation causing impaired awareness of hypoglycemia (IAH).^20^, ^21^ As many patients experience IAH, and classic glucose challenge tests may not always be accurate in identifying RH,^22^ the use of continuous glucose monitoring (CGM) has emerged as a much more effective and reliable tool in diagnosing and managing RH.^22^, ^23^, ^24^, ^25^, ^26^, ^27^, ^28^

The first‐line approach to treating RH is nutrition intervention and modification of eating patterns. Eating frequent meals every 3 h, avoiding fluids with meals, avoiding simple sugars in meals, consuming foods with a low glycemic index or low‐carbohydrate diets, and not consuming alcohol are recommended.^29^, ^30^, ^31^, ^32^ However, robust studies evaluating the actual impact of these approaches on glycemic control are limited.^30^ Pairing nutrition interventions with CGM could help evaluate the efficacy of these strategies in managing RH postesophagectomy.

This current study had two objectives. Firstly, CGM was used to accurately determine the incidence of symptomatic and asymptomatic RH events in a cohort of disease‐free patients postesophagectomy. Secondly, through detailed food and symptom diaries, the influence that dietary intake or specific eating patterns had on the incidence of an RH event was explored.

## MATERIALS AND METHODS

### Participant recruitment

Data were collected in the National Centre for Oesophageal and Gastric Cancer at St James's Hospital between October 2021 and March 2022. Ethical approval was obtained by the research ethics committee for Tallaght University Hospital and St James's Hospital [2020‐10 Chairman's Action (49)].

This study sought to recruit participants >12 months postesophagectomy. Consecutive patients who had an esophagectomy for EC from January 2018 to March 2021 with curative intent and were alive without evidence of disease recurrence for at least 12 to 36 months were eligible for recruitment. The exclusion criteria included previous gastrectomy procedure, presence or recurrence of cancer postesophagectomy, and a previous diagnosis of diabetes. Participants were identified through the hospital database and invited to attend an in‐person clinic, where informed consent to participate was obtained.

Clinicopathological details were extracted from participants records. Anthropometric measurements including height (cm) and weight (kg) were recorded following standard protocols.

### Glucose monitoring

All participants had a FreeStyle Libre (Abbott) CGM system applied to their upper arm as per manufacturer's instructions. This allowed for the recording of interstitial glucose concentrations every 15 min for a 7‐day period. Participants were given a FreeStyle Libre glucose reader to enable them to check interstitial glucose levels when desired and to facilitate transfer of data to the reader at 6 to 8‐h intervals. After the 7‐day period, glucose sensors and readers were returned to the center, and data were uploaded for analysis according to the manufacturer's instructions.

The RH events were identified as consecutive readings for ≥15 min 1 to 3 h postprandially with two levels of severity, level 1 being ≤70.2 mg/dl (3.9 mmol/L) and level 2 being ≤54 mg/dl (3 mmol/L).^33^

### Symptom recording

Over the 7‐day period, participants were required to record contemporaneously any symptoms relating to RH experienced during or after meals. This included abdominal pain, cramps, nausea, vomiting, bowel motions, dizziness, flushing, palpitations, sudden tiredness, shakiness, needing to lie down, headache, and sweating.^16^, ^17^, ^34^ If neuroglycopenic symptoms occurred within the time frame of RH, the event would be coded as a symptomatic event.

At the end of the 7‐day recording period, participants were asked to reflect on and summarize the severity and timing of specific symptoms they may have experienced after meals throughout the week while completing the modified Oesophageal Surgery in Cancer Patients: Adapt and Recovery Study (mOSCAR) questionnaire. This questionnaire assesses details of neuroglycopenic symptoms and determines if symptoms were resulting from early dumping syndrome (within 60 min of meal) or RH (>2 h after meal).

### Dietary intake analysis

Over the 7 days, participants completed a detailed food diary. Participants were given a tutorial to explain how to accurately document timing of eating, type of food consumed, and portion sizes. Nutrition data from each food diary were inputted into Nutritics (Educational version 5.93) to calculate the nutrition composition of each meal. Each diary was reviewed to explore compliance with specific recommended eating patterns. The following eating patterns were identified and coded: (1) having regular meals at least every 3 h, (2) avoiding fluids with meals, (3) avoiding simple sugars in meals, (4) and avoiding alcohol.^29^, ^30^

### Data analyses

All data were analyzed using IBM SPSS Statistics software (version 28.0.0.0). Mann‐Whitney *U* and Kruskal‐Wallis test were used to compare postprandial glucose readings between normoglycemic responses, asymptomatic RH events, and symptomatic RH events. To investigate the impact of dietary intake and eating patterns on RH events, independent samples *t* tests and chi‐square tests were performed. In all cases, *P* < 0.05 was considered statistically significant.

## RESULTS

### Participant characteristics

Participant demographic characteristics are displayed in Table 1. There were 32 participants in this study; over three quarters of participants were men (*n* = 28, 87.5%). The mean age of participants was 66.83 ± 9.78 years, with ages ranging from 46 to 84 years. Over 40% of the cohort were a healthy weight, with a body mass index (BMI), calculated as weight in kilograms divided by height in meters squared, of 18.5–24.9 (*n* = 14, 43.8%), and over half had a BMI >25 (*n* = 17, 53.1%). There was a mean weight reduction of 8.3 kg ± 8.7 (9.5% ± 10.1%) from time of surgery to time of recruitment. The mean time since surgery was approximately two and a half years and the majority of the esophagectomy procedures were Ivor Lewis procedures (*n* = 19, 73.1%). The most common medication taken was a proton pump inhibitor (*n* = 24, 75%), and four (12.5%) of the participants were taking high protein high calorie oral nutrition supplements.

**Table 1:** Participant demographic characteristics, oncological, surgical, and clinical details.

Demographic characteristics	Participants (*n* = 32)
Sex, *n* (%)	
Men	28 (87.5)
Women	4 (12.5)
Participant age upon recruitment,^a^ mean ± SD, years	66.83 ± 9.78
Age range of participants upon recruitment,^a^ *n* (%)	
Under 65 years	13 (50)
Over 65 years	13 (50)
Employment status,^b^ *n* (%)	
Working	19 (61.3)
Retired	12 (38.7)
Alcohol consumers at present, *n* (%)	
Yes	18 (56.3)
No	14 (43.7)
Current BMI range, mean ± SD, kg/m^2^	24.5 ± 0.63
Current BMI categories, *n* (%)	
Underweight	1 (3.1)
Healthy weight	14 (43.8)
Overweight	13 (40.6)
Obese	4 (12.5)
Weight change since surgery, mean ± SD, kg	−8.3 ± 8.7
Percentage weight change, mean ± SD, %	−9.5 ± 10.1
Oncological, surgical, and clinical details	
Tumor morphology,^a^ *n* (%)	
Adenocarcinoma	24 (92.3)
Squamous cell carcinoma	2 (7.7)
Type of surgery,^a^ *n* (%)	
Transhiatal esophagectomy	7 (26.9)
Ivor Lewis	19 (73.1)
Time since surgery,^a^ mean ± SD, months	24.58 ± 10.01
Current medications, *n* (%)	
Proton pump inhibitor	24 (75)
Pancrelipase	2 (6.3)
Domperidome	7 (21.9)
Colesevelam	0 (0)
Multivitamin and/or mineral	16 (50)
Oral nutrition supplement	4 (12.5)

Abbreviation: BMI, body mass index.

^a^ Data available from 26 participants.

^b^ Data available from 31 participants.

### Overall frequency and intensity of postprandial symptoms

Twenty‐eight of 32 participants completed the mOSCAR questionnaire, of which 96.4% reported experiencing at least one symptom postprandially (Table 2). Of these symptoms, 66.37% were reported ≤60 min after a meal, indicating symptoms of early dumping syndrome. The most common symptom experienced was sleepiness (67.9%), which was closely followed by abdominal cramps (64.3%).

**Table 2:** Frequency and timing of symptoms in patients from modified Oesophageal Surgery in Cancer: Adapt and Recovery Study dumping syndrome questionnaire.

Symptoms	Frequency reported	≤60 min	Timing ≥ 2 h	Time unknown
Sweating,^a^ *n* (%)	5 (17.9)	4 (80)	1 (20)	
Dizziness, *n* (%)	10 (35.8)	5 (50)	1 (10)	4 (40)
Palpitations, *n* (%)	6 (21.4)	4 (66.7)		2 (33.3)
Faint,^a^ *n* (%)	7 (25.1)	5 (71.4)		2 (28.6)
Nausea and/or vomiting,^a^ *n* (%)	12 (42.8)	8 (66.7)		4 (33.3)
Cramps,^a^ *n* (%)	18 (64.3)	12 (66.7)	4 (22.22)	2 (11.1)
Diarrhea,^b^ *n* (%)	15 (53.6)	10 (66.7)	1 (6.7)	4 (26.67)
Shaky, *n* (%)	11 (39.2)	8 (72.7)	2 (18.2)	1 (9.1)
Difficulty concentrating, *n* (%)	10 (35.8)	6 (60)	2 (20)	2 (20)
Sleepiness,^c^ *n* (%)	19 (67.9)	13 (68.4)	3 (15.8)	1 (5.3)

^a^ Data available from 27 participants.

^b^ Data available from 26 participants.

^c^ Data available from 25 participants.

### Glucose distribution 3 h postprandially

**Figure 1:**
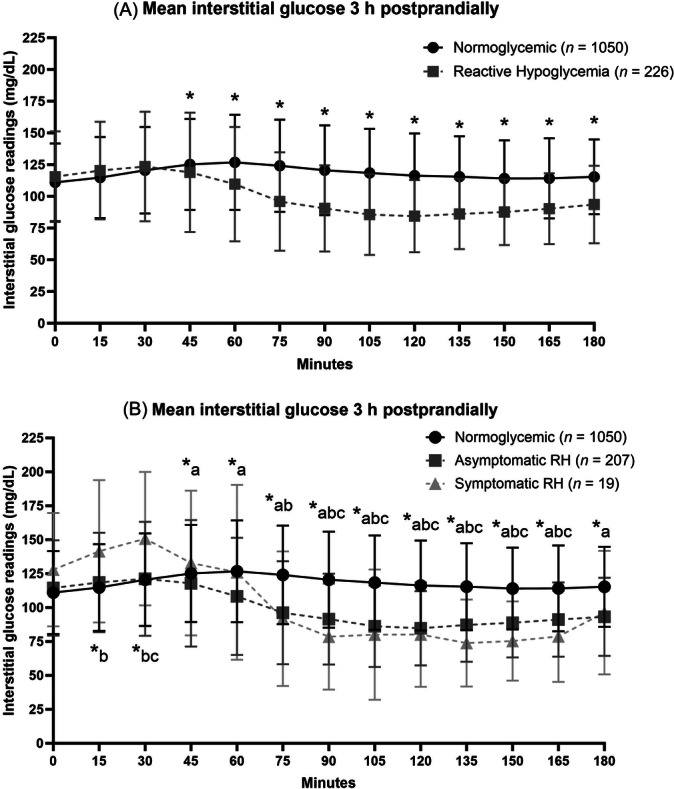
Line chart showing mean interstitial glucose readings 3 h postprandially comparing normoglycemic response with RH response (A) and comparing normoglycemic response with asymptomatic RH and symptomatic RH response (B). * *P* ≤ 0.05 between normoglycemic response and any RH event. *a, *P* ≤ 0.05 between normoglycemic response and asymptomatic RH event. *b, *P* ≤ 0.05 between normoglycemic response and symptomatic RH event. *c, *P* ≤ 0.05 between asymptomatic RH event and symptomatic RH event. RH, reactive hypoglycemia.

Figure 1A represents the distribution of mean interstitial glucose readings over 3 h postprandially, separated into readings from meals followed by no hypoglycemic events and meals followed by an RH event. Divergence was observed after 45 min, with levels in RH events falling (118.8 vs 126.0 mg/dl) and continuing to be significantly lower than normoglycemic responses at all timepoints up to 3 h postprandially (*P* < 0.05 in all cases). When the RH events were separated into asymptomatic and symptomatic RH, a different pattern emerged (Figure 1B). Within the first 30 min postprandially, symptomatic RH was associated with significantly higher interstitial glucose levels compared with normoglycemic and asymptomatic RH responses. After 30 min, interstitial glucose levels in symptomatic RH began falling and fell significantly below normoglycemic response levels from 75 min (91.8 vs 124.2 mg/dl) and asymptomatic RH levels from 90 min (79.2 vs 91.8 mg/dl), remaining significantly lower until 165 min postprandially (*P* < 0.05 in all cases).

### RH events and RH symptoms

Table 3 illustrates the frequency of RH events. There were 1276 meals investigated, and 17.7% (*n* = 226) of these were followed by an RH event with most of these events being level 1 RH events (*n* = 194, 85.8%). Nearly all participants (*n* = 31, 96.9%) experienced at least one RH event. Only 8.4% of the RH events were symptomatic, and over half of these were level 2 RH events (*n* = 10, 53%). A total of 12 participants experienced a symptomatic RH event (37.5%). The most common symptom was weakness and/or dizziness (*n* = 7, 36.8%).

**Table 3:** Frequency of postprandial reactive hypoglycemic events and symptoms experienced.

Total meals evaluated (*n* = 1276)	Frequency	Percentage of meals
Reactive hypoglycemic events		
No reactive hypoglycemic events	1050	82.3
Reactive hypoglycemic events	226	17.7
Level 1 events	194 (85.8% of RH events)	15.2
Level 2 events	32 (14.2% of RH events)	2.5
Symptomatic RH events	19 (8.4% of RH events)	1.5
Level 1 events	9 (47% of symptomatic RH events)	0.7
Level 2 events	10 (53% of symptomatic RH events)	0.7
Reported symptoms during symptomatic RH event		
Fatigue	6 (31.6% of symptoms)	0.5
Weakness and/or dizziness	7 (36.8% of symptoms)	0.5
Cramps	2 (10.5% of symptoms)	0.2
Dumping	2 (10.5% of symptoms)	0.2
Nausea	2 (10.5% of symptoms)	0.2

Abbreviaton: RH, reactive hypoglycemia.

### Nutrition composition of meals and RH events

Tables 4 and 5 demonstrate the difference in dietary content of meals that had no hypoglycemic event compared with meals that had an RH event (Table 4) and of meals that had a symptomatic vs an asymptomatic RH event (Table 5). There was a significantly higher mean total carbohydrate (*P* = 0.036), fiber (*P* = 0.020), and sugar (*P* = 0.048) content in meals followed by an RH event. There was no significant difference when these variables were considered as a percentage of total energy. There was a trend toward lower protein and fat contents as a percentage of total energy in meals followed by an RH event (*P* = 0.071 and *P* = 0.064, respectively).

**Table 4:** Comparison of the mean dietary content of meals followed by no hypoglycemic event vs meals followed by a reactive hypoglycemic event.

Variable	No hypoglycemia (*n* = 1050)	Reactive hypoglycemia (*n* = 226)	*P* value
Energy, mean (±SD), kcal	302 (±241.23)	323 (±216.04)	0.237
Carbohydrate, mean (±SD), g	31.7 (±23.64)	35.3 (±22.57)	0.036
Energy, mean (±SD), %	47.63 (±24.70)	49.32 (±26.87)	0.365
Protein, mean (±SD), g	13.03 (±19.98)	13.25 (±15.58)	0.871
Energy, mean (±SD), %	16.03 (±11.89)	14.46 (±10.38)	0.071
Fat, mean (±SD), g	12.8 (±12.8)	13.1 (±12.25)	0.710
Energy, mean (±SD), %	35.75 (±24.03)	32.56 (±17.58)	0.064
Fiber, mean (±SD), g	3.15 (±4.99)	4.11 (±4.13)	0.020
Energy, mean (±SD), %	4.25 (±7.45)	5.13 (±11.22)	0.152
Sugar, mean (±SD), g	10.96 (±11.59)	12.65 (±11.63)	0.048
Energy, mean (±SD), %	21.30 (±26.15)	21.99 (±24.29)	0.717

**Table 5:** Comparison of the mean dietary content of meals followed by asymptomatic RH events vs meals followed by symptomatic RH events.

Variable	Asymptomatic RH event (*n* = 207)	Symptomatic RH event (*n* = 19)	*P* value
Energy, mean (±SD), kcal	321 (±214.83)	257 (±240.5)	0.219
Carbohydrate, mean (±SD), g	35.27 (±22.52)	30.18 (±21.97)	0.345
Energy, mean (±SD), %	49.02 (±22.13)	53.23 (±18.41)	0.421
Protein, mean (±SD), g	13.34 (±15.67)	9.08 (±10.59)	0.247
Energy, mean (±SD), %	14.37 (±10.39)	13.12 (±10.04)	0.614
Fat, mean (±SD), g	12.79 (±11.87)	10.47 (±14.4)	0.422
Energy, mean (±SD), %	31.66 (±17.9)	30.4 (±15.69)	0.766
Fiber, mean (±SD), g	4.2 (±8.09)	3.02 (±4.17)	0.531
Energy, mean (±SD), %	5.45 (±11.49)	3.55 (±2.89)	0.474
Sugar, mean (±SD), g	12.62 (±11.45)	11.51 (±10.19)	0.683
Energy, mean (±SD), %	22.75 (±24.50)	28.79 (±23.7)	0.302

Abbreviation: RH, reactive hypoglycemia.

Nutrient composition of meals did not appear to influence the occurrence of symptomatic RH events vs asymptomatic RH events.

### Modifiable eating patterns and RH events

Table 6 describes the frequency of meals with and without compliance with recommended eating patterns. From the 1276 meals investigated, only 16.1% complied with all recommended eating patterns specific to this study. The most common guideline not followed was consuming fluids with meals, with over half of the meals being consumed with fluids (*n* = 699, 65.3%).

**Table 6:** Frequency of compliance with recommended modifiable eating patterns.

Eating patterns	*n* (%)
Compliance with recommended eating patterns (*n* = 1276 meals evaluated)	
Compliant with recommended eating patterns	205 (16.1)
Not compliant with recommended eating patterns	1071 (83.9)
Specific eating patterns (*n* = 1071 noncompliant meals)	
>3 h between meals	583 (54.4)
Fluid with meals	699 (65.3)
Simple sugars with meals	472 (44.1)
Consuming alcohol	65 (6.1)

Table 7 illustrates the incidence of RH events following meals not compliant with specific recommended eating guidelines, and Table 8 separates RH events into symptomatic vs asymptomatic RH events. There was a significantly higher frequency of RH events when meals were eaten >3 h apart (*P* = 0.019) and when meals were consumed with alcohol (*P* = 0.008). When investigating simple sugars with meals and the incidence of RH events, it appeared that RH events occurred more frequently following meals with more simple sugars (*P* = 0.059).

**Table 7:** Comparison of noncompliance to recommended eating patterns and incidence of reactive hypoglycemia events.

Variable	No hypoglycemia (*n* = 1050)	Reactive hypoglycemic (*n* = 226)	*P* value
>3 h between meals, *n* (%)	477 (45.4)	120 (53.1)	0.019
Fluid with meals, *n* (%)	579 (55.1)	130 (57.5)	0.483
Simple sugars with meals, *n* (%)	376 (35.8)	104 (46)	0.059
Consuming alcohol, *n* (%)	45 (4.3)	21 (9.3)	0.008

*Note*: Chi‐square test comparing frequency of non hypoglycemic events and reactive hypoglycemic events following non‐compliance with recommended eating patterns.

**Table 8:** Comparison of noncompliance to recommended eating patterns and incidence of asymptomatic vs symptomatic RH events.

Variable	Asymptomatic RH event (*n* = 207)	Symptomatic RH event (*n* = 19)	*P* value
>3 h between meals, *n* (%)	104 (50.2)	7 (36.8)	0.477
Fluids with meals, *n* (%)	123 (59.4)	7 (36.8)	0.186
Simple sugars with meals, *n* (%)	97 (46.9)	7 (36.8)	0.378
Consuming alcohol, *n* (%)	20 (9.7)	1 (5.3)	0.970

*Note*: Chi‐square test comparing frequency of asymptomatic and symptomatic RH events following non‐compliance with recommended eating patterns.

Abbreviation: RH, reactive hypoglycemia.

Eating patterns did not appear to influence the incidence of symptomatic RH events vs asymptomatic RH events.

## DISCUSSION

To our knowledge, this is the first study to use CGM technologies and detailed food and symptom diaries to comprehensively evaluate postprandial glucose control in patients postesophagectomy.

### Incidence of symptomatic and asymptomatic RH in this cohort

When considering all glucose readings from these participants, 4.58% of readings were below range, which is 2.1% more than that of population reference values^33^ (Supplementary Table 1). For the 1276 meals investigated, 226 (17.7%) were followed by an RH event. Interestingly, only 8.4% of events were reported as symptomatic, indicating the high prevalence of IAH in these patients. Overall, there was a very low incidence of level 2 RH events (*n* = 32, 14.2%); however, when considering just symptomatic RH events (*n* = 19, 8.4%), there was a greater proportion of level 2 events (*n* = 10, 53%). This suggests there may be a threshold for glucose levels below which symptoms begin, which has been previously observed.^35^

It was evident in this study that symptoms alone cannot detect an RH event. Upon investigation of the neuroglycopenic symptoms these patients experienced, many of them appeared to result from early dumping syndrome, as evidenced by the results from the mOSCAR dumping syndrome questionnaire. This questionnaire showed that of the neuroglycopenic symptoms reported, only 12.4% occurred >2 h postprandially, indicating they were related to RH.^36^ This result is consistent with the existing literature that patients postesophagectomy more commonly experience symptoms of early dumping syndrome rather than late dumping syndrome or RH^19^, ^37^, ^38^ and further highlights the potential role of CGM use in these patients. It should be noted, however, that not all participants completed the mOSCAR questionnaire. The results may not be entirely accurate because it was completed at the end of a 7‐day period, meaning participants may have forgotten the details of the symptoms they experienced. Further, it does not consider symptoms experienced 1–2 h after a meal, only <1 h and >2 h after a meal.

When examining the pattern of postprandial glucose readings, a clear divergence is observed after 30 min between the normoglycemic response and RH events, which continued for the duration of postprandial observation. Interestingly, when RH events were separated into asymptomatic and symptomatic RH, the symptomatic RH events demonstrated a “glucose spike” immediately postprandially before sloping downwards. The current doctrine of RH physiology is that there is a glucose spike because of “dumping” into the small intestine followed by an insulin spike, which causes the abnormally low glucose in hypoglycemia.^39^, ^40^ It may be that this particular pattern of glucose excursion is associated with greater neuroglycopenic symptoms and thus more symptomatic RH events. However, in the current study, results may be skewed because of the low total number of symptomatic events reported (*n* = 19). Further studies should consider measuring the insulin or glucagon‐like peptide 1 (GLP‐1) levels of participants alongside glucose to further elucidate the pathophysiology of RH.^15^, ^39^, ^40^, ^41^

### Influence of dietary intake on RH events

In this current study, meals higher in total carbohydrate and sugar were associated with RH events. The carbohydrate and sugar content of meals is fundamental in managing postprandial glucose levels in RH.^38^, ^42^ In patients postgastric bypass experiencing RH, it has been recommended to limit carbohydrate intake to 30 g for meals and 15 g for snacks, resulting in an intake of 90–130 g/day to effectively manage glycemic responses. However, this low intake may not be suitable for patients postesophagectomy who, as observed in the current study, may struggle with healthy weight maintenance.^30^, ^32^ Interestingly, when carbohydrate and sugar were considered as a percentage of total energy, they did not have an influence on RH events, nor did the presence of protein or fat significantly influence the incidence of RH. The role that protein and fat have in attenuating glycemic response is based on their influence on gut hormone signaling (gastric inhibitory polypeptide, GLP‐1, and insulin) and gastric emptying.^43^ However, the actual effectiveness of increasing the protein or fat content of meals as an RH management strategy, specifically postesophagectomy, has not been reported. The findings of the current study suggest that the absolute carbohydrate content of the meal has more influence on postprandial glycemia in these patients, rather than the energy distribution of the meal.

The observation that meals with a higher fiber content were associated with RH events is interesting and, in many ways, counterintuitive. Dietary fiber, especially soluble dietary fiber, has been shown to delay gastric emptying, and both soluble and insoluble fiber are recommended to control glucose levels.^44^ The observations in this study may be a result of the higher fiber meals also having higher carbohydrate content. It should be noted that in the current study, the glycemic index and/or glycemic load of meals was not considered, nor was the food texture, speed of ingestion, or sequencing of nutrient consumption within a meal, all factors which may be altered in individuals postesophagectomy.^30^, ^43^ Further research is needed to determine whether the standard nutrition advice of consuming mixed meals and including fiber^30^, ^32^ are truly effective at managing RH in patients postesophagectomy and should also consider the role of interindividual variability in glycemic responses.^45^, ^46^

### Influence of specific eating patterns on RH events

It is recommended for patients with RH to have small frequent meals every 3 h.^15^, ^29^, ^32^ Despite the widespread acceptance of this recommendation as part of nutrition management, there is little evidence that it is effective in preventing RH. In this study, nonadherence to this guideline was associated with an increase in RH events, supporting its validity in managing RH postesophagectomy.

Similarly, publications have recommended that alcohol be avoided in patients who experience RH without reference to any specific clinical studies.^29^, ^32^, ^38^ In the current study, consuming alcohol with meals significantly increased the incidence of RH events. This concurs with a cross‐sectional study by Oba‐Yamamoto et al, in which the authors found the incidence of RH was more prevalent with an alcohol and glucose load than just a glucose load alone.^47^ It should be noted that in the current study, given the small overall number of episodes of alcohol consumption, subgroup analysis of the impact of different types of alcohol, mixers, or nutrient composition of meals on RH was not explored and warrants further research.^48^, ^49^

In the current study, neither nutrient content nor eating patterns appeared to influence the incidence of symptomatic vs asymptomatic RH events. This is an interesting finding and further highlights the potential role of CGM use in these patients.^22^, ^23^, ^35^, ^50^ Patients who experience IAH may have altered perceptions of their illness and ignore recommended behaviors to prevent RH events because they are unaware they are having them. CGM may help encourage effective behavior change and self‐management.^35^, ^51^

### Strengths and limitations

The design of this study allowed for robust data collection. The use of CGM and gold standard 7‐day food diaries resulted in a combined total of 21,504 glucose readings and 1276 meals being analyzed, allowing a comprehensive evaluation of postprandial glucose levels, dietary intake, and symptoms.

The limitations of this study include the use of self‐reported dietary intake, which may result in inaccuracies and underreporting of energy, fat, and simple carbohydrate intake.^52^, ^53^ Furthermore, whereas dietary intake was recorded, the sequence of nutrient consumption within a meal was not, and this may influence glycemic responses.^54^, ^55^ It should also be noted that there was no subanalysis of timing of symptoms postprandially within the 1–3 h window, and the mOSCAR tool comparing early dumping vs RH was completed retrospectively at the end of the week.

Considering the demographic characteristics of participants in this study, the division of sex was 87.5% men to 12.5% women, which differs from Irish EC statistics of approximately 70% men to 30% women.^56^

The research findings presented require further validation through intervention studies with expanded sample sizes, refined dietary pattern analyses, and exploration of the long‐term effects of dietary interventions.

## CONCLUSION

The findings from this study confirm that the carbohydrate content in meals significantly influenced the occurrence of RH events and that eating patterns, specifically having frequent meals, and avoiding alcohol with meals prevented RH. Ensuring all EC survivors have access to dietetic support to understand and self‐manage their risk of RH is imperative. Furthermore, this study has demonstrated the value of CGM as a supportive tool in identifying RH in this patient cohort.

## AUTHOR CONTRIBUTIONS

Claire L. Donohoe and John V. Reynolds contributed to the conception and design of the research. Michelle Fanning and Niamh Ni Mhaoinigh contributed to the design of the research. Rachel O'Kelly, Elaine Quigley, Katie Byrne, Mistura A. Kareem, Paul Healy, Michelle Fanning, Claire L. Donohoe, and Suzanne L. Doyle contributed to the acquisition and analysis of the data. Rachel O'Kelly and Suzanne L. Doyle contributed to the interpretation of the data. Rachel O'Kelly and Suzanne L. Doyle drafted the manuscript. All authors critically revised the manuscript, agree to be fully accountable for ensuring the integrity and accuracy of the work, and read and approved the final manuscript.

## CONFLICT OF INTEREST STATEMENT

None declared.

## Supporting information

NCP‐2025‐01‐036.
