# Supplementary material for: Influence of dietary intake and eating patterns on reactive hypoglycemic events in patients postesophagectomy: A prospective observational study using continuous glucose monitoring
Source: Nutr Clin Pract. 2025 Sep 7;41(3):880–91. doi: 10.1002/ncp.70022 (PMC13193482; doi:10.1002/ncp.70022)
Supplement: Supplementary file 1 — NCP‐2025‐01‐036. [file NCP-41-880-s001.docx]

Supplemental Table 1: Interstitial Glucose Metrics

|  | Study Population (n=32) | **Population Reference Values33** |
| --- | --- | --- |
| **Time in range** (% of readings)  72-180mg/dL (4.0-10.0mmol/L) | 91.8 ± 5.03 | 94.8 |
| **Time below range** (% of readings)  54-70.2mg/dL (3.0-3.9mmol/L)  <54mg/dL (<3mmol/L) | 4.2 ± 3.91  0.38 ± 0.45 | 2.1  0.4 |
| **Time above range** (% of readings)  181.8-250.2mg/dL (10.1-13.9mmol/L)  >250.2mg/dL (>13.9mmol/L) | 3.3 ± 2.61  0.27 ± 0.55 | *  * |

* *Data unavailable*
